# Associations between physical activity and cardiorespiratory fitness with vascular health phenotypes in older adults: a cross-sectional study

**DOI:** 10.3389/fphys.2023.1096139

**Published:** 2023-05-15

**Authors:** Maria Karolina Ferreira de Sousa, Raíssa de Melo Silva, Yuri Alberto Freire, Gabriel Costa Souto, Marcyo Câmara, Ludmila Lucena Pereira Cabral, Geovani Araújo Dantas Macêdo, Eduardo Caldas Costa, Ricardo Santos Oliveira

**Affiliations:** ^1^ Department of Physical Education, Federal University of Rio Grande do Norte, Natal, RN, Brazil; ^2^ Department of Physical Education, ExCE Research Group, Federal University of Rio Grande do Norte, Natal, RN, Brazil; ^3^ Graduate Program in Health Sciences, Federal University of Rio Grande do Norte, Natal, Brazil; ^4^ INTEGRA—Integrative Physiology, Health, and Performance Research Group, Federal University of Rio Grande do Norte, Natal, RN, Brazil

**Keywords:** intima-media thickness, pulse wave velocity, 6-min walk test, accelerometry, cardiovascular diseases

## Abstract

**Objective:** We investigated the associations between physical activity (PA) and cardiorespiratory fitness (CRF) with vascular health phenotypes in community-dwelling older adults.

**Methods:** This cross-sectional study included 82 participants (66.8 ± 5.2 years; 81% females). Moderate-to-vigorous physical activity (MVPA) was assessed using accelerometers, and CRF was measured using the distance covered in the 6-min walk test (6MWT). The vascular health markers were as follows: i) arterial function measured as aortic pulse wave velocity (aPWV) estimated using an automatic blood pressure device; and ii) arterial structure measured as the common carotid intima-media thickness (cIMT). Using a combination of normal cIMT and aPWV values, four groups of vascular health phenotypes were created: normal aPWV and cIMT, abnormal aPWV only, abnormal cIMT only, and abnormal aPWV and cIMT. Multiple linear regression was used to estimate the beta coefficients (*β*) and their respective 95% confidence intervals (95% CI) adjusting for BMI, and medication for diabetes, lipid, and hypertension, sex, age, and blood pressure.

**Results:** Participants with abnormal aPWV and normal cIMT (β = −53.76; 95% CI = −97.73—−9.78 m; *p* = 0.017), and participants with both abnormal aPWV and cIMT (β = −71.89; 95% CI = −125.46—−18.31 m; *p* = 0.009) covered less distance in the 6MWT, although adjusting for age, sex and blood pressure decreased the strength of the association with only groups of abnormal aPWV and cIMT covering a lower 6MWT distance compared to participants with both normal aPWV and cIMT (β = −55.68 95% CI = −111.95–0.59; *p* = 0.052). No associations were observed between MVPA and the vascular health phenotypes.

**Conslusion:** In summary, poor CRF, but not MVPA, is associated with the unhealthiest vascular health phenotype (abnormal aPWV/cIMT) in older adults.

## 1 Introduction

Ageing has negative effects on the cardiovascular system, including detrimental changes in arterial function, stiffness, and structure, which can lead to cardiovascular disease (Kobayashi et al., 2004; Costantino et al., 2016). Aortic pulse wave velocity (aPWV) measures arterial stiffness (Townsend et al., 2015) and function (Kinlay et al., 2001; Wilkinson et al., 2002), whereas ultrasound imaging assessment of the common carotid intima-media thickness (cIMT) provides a measure of arterial structure (Safar, 2010). Both increased aPWV and cIMT predict cardiovascular disease morbidity and mortality (Blacher and Safar, 2005; Lorenz et al., 2007; Safar, 2010; Polak et al., 2011). Expert consensuses suggest using aPWV >10 m/s and cIMT above the 75th percentile for sex, age, and race as cut-off values in clinical practice (Stein et al., 2008; Van Bortel et al., 2012) because of their association with an increased incidence of cardiovascular diseases (Sequí-Domínguez et al., 2020; van den Oord et al., 2013; Vlachopoulos et al., 2010), which are the leading causes of death among older adults (WHO, 2020).

In addition to the prognostic value of aPWV and cIMT, combining these vascular health markers can increase their predictive capacity beyond that of each measure alone. Previous research has indicated that a combination of aPWV >10.2 m/s and cIMT >1 mm is associated with poorer cardiac function (Tzortzis et al., 2010), particularly among older adults, combining these markers strongly predicts vascular events in a longitudinal follow-up beyond each index alone (Nagai et al., 2013). As such, it is possible to combine aPWV and cIMT and create distinct “vascular health phenotypes,” with healthiest and unhealthiest phenotypes defined as having normal and abnormal values for both measures, respectively. Identifying lifestyle behaviour and individual characteristics associated with specific vascular health phenotypes is important to aid preventative strategies focused on cardioprotective effects in older adults (Ross et al., 2016).

Cumulative evidence indicates that physical activity (PA) and cardiorespiratory fitness (CRF) have positive impacts on aPWV (Kodama et al., 2009; Vlachopoulos et al., 2010) and cIMT (Luedemann et al., 2002; Park et al., 2017; Germano-Soares et al., 2018; Deiseroth et al., 2019; Câmara et al., 2020; Lee et al., 2020; Vandercappellen et al., 2020). However, studies investigating the association between PA and CRF with vascular health phenotypes, including the combination of aPWV and cIMT, are lacking. For instance Gomez-Marcos et al. (2014), found no association between objectively measured PA and cIMT or aPWV in a sample of 55-year-old adults, although they did not combine vessel outcomes and investigate CRF. In terms of CRF Hinrichs et al., (2022), demonstrated that the distance covered in the 6-min walk test (6MWT) was not related to aPWV in older adults, whereas a previous study reported an inverse association between aPWV and the distance covered in a 400-m walk (ALBIN et al., 2020). These findings suggest that although the distance covered in the 6MWT is associated with general health in older adults (Bautmans et al., 2004), its relationship with vascular health markers remains debatable. Importantly, the 6MWT is a measure of CRF in older adults with or without comorbidities (Bean et al., 2002), has prognostic value (Agarwala and Salzman, 2020), responds to aerobic training (Bouaziz et al., 2018), and is easy to use in clinical practice, as recommended by the American Heart Association (Ross et al., 2016). Considering that both aPWV and cIMT are independent predictors of major cardiovascular disease events (Terentes-Printzios et al., 2017) and that these markers reflect different aspects of vascular health (Costantino et al., 2016), this study aimed to investigate the associations between PA, CRF, and vascular health phenotypes in community-dwelling older adults. We hypothesised that both low moderate-vigorous PA (MVPA) and CRF would be associated with the unhealthiest vascular health phenotype, characterised by abnormal aPWV/cIMT.

## 2 Materials and methods

### 2.1 Participants

The data presented in this study were obtained from older adult participants of an ongoing longitudinal investigation. Community-dwelling older adults aged 60–80 years were invited to participate in this project. The criterion for defining older adults as >60 years was based on the World Health Organization definition (Tramujas Vasconcellos Neumann and Albert, 2018). The recruitment was performed using advertisements on local radio, social networks, older adult community centres, primary attention clinics, and universities. The eligibility criteria were as follows: 1) no history of CVD and major adverse cardiovascular events, such as stroke, myocardial infarction, peripheral arterial diseases, and others; 2) no musculoskeletal injury limiting the ability to perform PA; and 3) systolic blood pressure (BP) < 160 mmHg, diastolic BP < 105 mmHg (Barroso et al., 2021), and fasting glucose <250 mg/dL (Sociedade Brasileira de Diabetes, 2019). The participants were informed of the study procedures and provided written consent to participate. The study was approved by the institutional Ethics Committee (protocol nº 2.603.422/2018).

### 2.2 Study design

This study is reported in accordance with the STROBE statement (von Elm et al., 2007). The data were collected between June 2018 and December 2019. The present study used data allowing for the investigation of associations between exposures (MVPA and 6MWT) and outcomes (aPWV and cIMT). Participants reported to the laboratory in the morning on two separate days, 1 week apart. On the first day, they were informed of the study procedures and gave assent to participate. A 12-h fasting blood sample was collected to obtain the participants’ metabolic profiles. Blood pressure (BP), arterial function, and structure were also measured. The participants were then allowed to eat breakfast before completing the sociodemographic questionnaire. At the end of the first day, the participants received accelerometers for PA assessment. On the second day, the participants returned the accelerometers and performed the 6MWT.

### 2.3 Exposures

#### 2.3.1 Physical activity

Triaxial accelerometers were used to obtain PA levels (ActiGraph GT3X, ActiGraph LLC, Pensacola, United States). The participants wore accelerometers on their right hip for 7 days. The devices were worn for the entire duration of the day, except for water activities such as showering and swimming. Participants also received a diary to note the time they took off the device as well as the time they went to sleep and wake up. Accelerometers were set to record acceleration at 60 Hz and 60 s epochs were used. Non-wearing time was defined according to (Choi et al., 2011) as periods of ≥90 consecutive minutes of zero counts, with a tolerance of ≥100 counts/min for up to 2 min. A valid PA assessment was considered when participants wore the device 10 h or more for at least 4 days, including one weekend day (Trost et al., 2005). Accelerometer-based PA measures were analysed from the weighted average of valid weekdays and weekends using the software ActiLife, version 6.13.3.2. The acceleration cut-offs proposed by Freedson et al. (1998) were used to obtain MVPA when counts per minute were ≥1,952.

#### 2.3.2 6-min walk test

The 6MWT was used to measure CRF (Rikli and Jones, 2013). Volunteers were instructed to cover the longest possible distance during a 6-min period while being verbally encouraged and informed about the time elapsed at the end of each minute. Participants were allowed to rest during the test, and a 1-min cool-down period of light walking occurred at the end. The 6MWT is a reliable and valid method for assessing CRF in community-dwelling older adults (Rikli and Jones, 1998; Rikli and Jones, 1999), and is endorsed by the American Heart Association (Ross et al., 2016). A previous investigation has demonstrated an *R* ^2^ value > 0.85 for the association between VO_2_max obtained and predicted with the 6MWT for both males and females (Mänttäri et al., 2018).

### 2.4 Outcomes

#### 2.4.1 Common carotid intima-media thickness

Using an ultrasound device (GE, Vivid I^®^, California, United States) a trained sonographer obtained images of the left common carotid artery (GE, Vivid I^®^, California, United States), following published guidelines (Touboul et al., 2012). A linear array transducer (10 MHZ) was used to obtain longitudinal images of the common carotid artery, approximately 2 cm from the carotid bulb. Images with clear definitions of the near and far walls of the artery were used to measure the cIMT at end diastole. The far-wall intima-lumen and media-adventitia interfaces were identified, and a total of six manual measurements were completed. The average of the six measurements was used as the cIMT in micrometres (µm). During the assessment, participants were in a supine position with their neck extended and their head tilted at ∼45° degrees.

#### 2.4.2 Aortic pulse wave velocity

Arterial stiffness was obtained as the aPWV. For this, an automatic BP device (Dyna-Mapa; Cardios; São Paulo, Brazil) was used. This device is a version of the Mobil-O-Graph 24 h Pulse Wave Analysis Monitor (IEM, Stolberg, Germany). The equipment measures the oscillometric waveforms at the brachial artery, and with a mathematical ARCSolver algorithm (Austrian Institute of Technology, Vienna, Austria), which uses pulse wave analysis and wave separation parameters, the aPWV was obtained (Wassertheurer et al., 2008). This pulse wave analysis has been validated against invasive intra-aortic catheter (Hametner et al., 2013) and applanation tonometry (Weber et al., 2011; Weiss et al., 2012; Hoshide et al., 2018). Measurements were performed in the left arm following a 10-min period of supine rest in a temperature-controlled room (24°C–26°C). The participants were asked not to speak during data collection. Per manufacturer’s instructions, four measurements were collected with intervals of 1-min being the first used to calibrate the device, and the average of the following 3 used as aPWV in m/s. Recent investigations have shown that the aPWV assessment used in the present study is associated with steps per day (Cabral et al., 2021) and frailty (Macêdo et al., 2022) in older adults.

#### 2.4.3 Vascular health phenotype

The participants were divided into four groups of vascular health according to a combination of aPWV and cIMT. It is important to note that in this study, cIMT and aPWV exhibited a poor and nonsignificant correlation (r = 0.03), indicating that they offer complementary information about vascular health. To define the vascular health phenotypes, we used a 10 m/s cut-off as increased aPWV and being above the 75th percentile for sex, age, and race based on the Brazilian population values of cIMT (Santos et al., 2014). The 10 m/s cut-off was considered based on the consensus of the European Society of Hypertension (Van Bortel et al., 2012). Additionally, a recent meta-analysis showed that values >9.9 m/s are associated with a higher incidence of cardiovascular and all-cause mortality (Sequí-Domínguez et al., 2020). Cut-off values for increased cIMT were based on the Brazilian Society of Cardiology (Santos et al., 2014), which follows the Consensus Statement from the American Society of Echocardiography (Stein et al., 2008). Based on these cut-offs, the participants were divided into the following groups:

• Normal vascular function and structure (aPWV <10 m/s and cIMT <75th percentile for sex, age, and race)
• Normal vascular function and abnormal structure (aPWV <10 m/s and cIMT >75th percentile for sex, age, and race)
• Abnormal vascular function and normal structure (aPWV >10 m/s and cIMT <75th percentile for sex, age, and race)
• Abnormal vascular function and structure (aPWV >10 m/s and cIMT >75th percentile for sex, age, and race)

### 2.5 Confounders

In this investigation the following confounders were used: medication for diabetes, dyslipidaemia, and BP, obtained from structured questionnaires; age and sex; body mass index (BMI) obtained as kilograms divided by metres squared; and systolic and diastolic brachial BP obtained using an automatic oscillometric method (Omron HEM-780-E, Kyoto, Japan) following a 10-min of sitting rest period (Malachias et al., 2016). Additionally, for participant characterisation, total cholesterol and fasting glucose were obtained using commercially available kits (Labtest^®^, Diagnostic Labtest-SA, São Paulo, Brazil), and the percentage of participants with hypertension, diabetes, and dyslipidaemia as well as the percentage of participants meeting current physical activity guidelines of 150 min per week of MVPA (Izquierdo et al., 2021).

### 2.6 Statistical analysis

Continuous data are presented as mean ± standard deviation and categorical data are presented as absolute and relative frequencies. The association between MVPA and 6MWT with vascular health phenotypes was determined using multiple linear regression. Beta coefficients were adjusted for BMI and medication for diabetes, lipid, and hypertension in the first model (model 1), and further adjusted for sex, age, systolic, and diastolic BP in the second model (model 2). Although age, sex and BP were used to create the vascular health phenotypes and to estimate aPWV, Model 2 also considered these variables as confounders because they are known to influence aPWV (Papaioannou et al., 2016). In both models, MVPA was log10 transformed to assure a normal distribution of residuals.

Multiple linear regression adjusted for BMI, and medication to control diabetes, lipid and hypertension, age, sex, and systolic and diastolic BP was also used to investigate whether a dose-response exists for quartiles of MVPA and 6MWT on aPWV and cIMT. Model assumptions were checked using the Shapiro-Wilk’s test, Durbin Watson test, and normal distribution of residuals. Multicollinearity was checked using variance inflation factor (VIF) < 3 and tolerance <0.1 for each variable inserted in the model. Analyses were conducted in RStudio v 1.4.1717^©^, with significance set at *p* < 0.05.

## 3 Results

**FIGURE 1:**
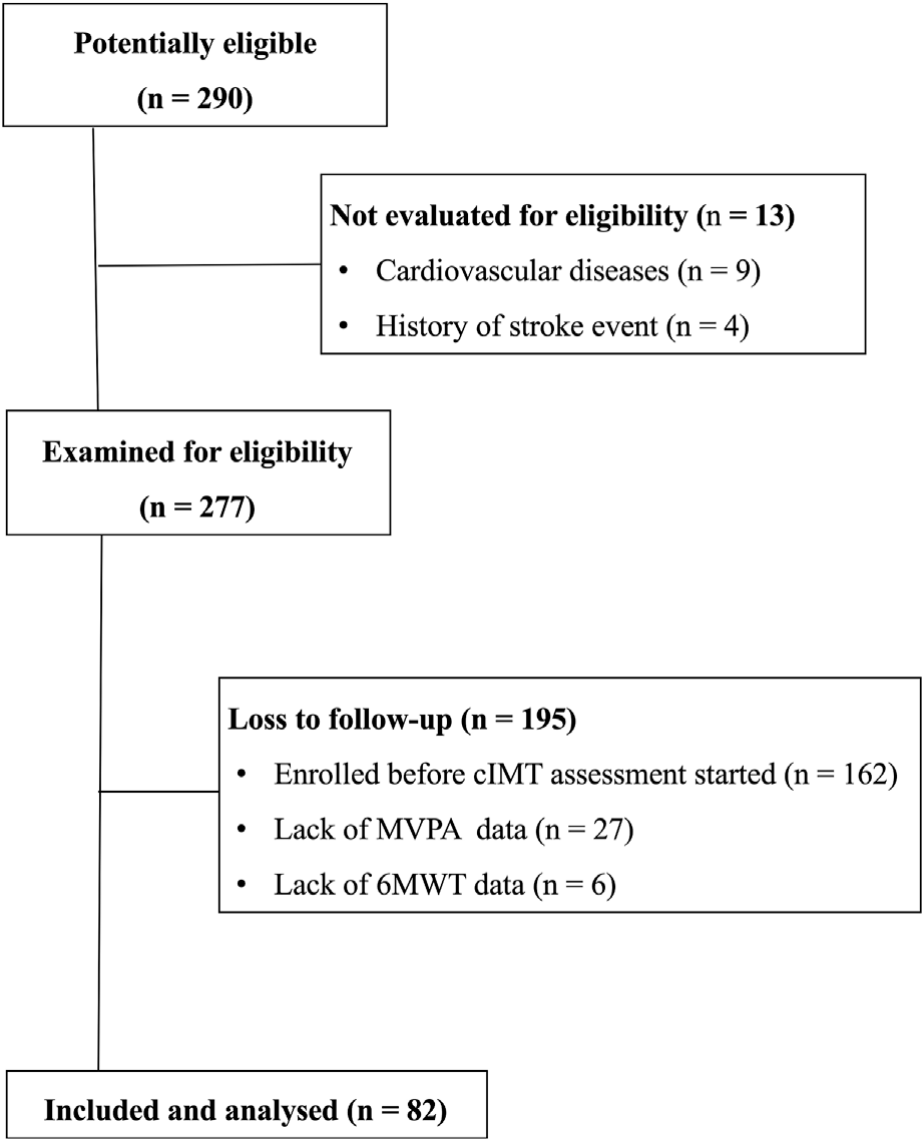
Flow chart with the included participants.

The flowchart of the participants included in the analysis is shown in Figure 1. Of 290 volunteers, 277 had their aPWV assessed. Of these 277 participants, 162 did not have an assessment of cIMT, as they enrolled in the investigation before cIMT assessments were in place. Of the 115 participants who underwent both cIMT and aPWV assessments, 27 did not complete the objective PA assessment, and 6 did not complete the 6MWT. As a result, 82 participants (66 women) were included in the present analysis. The participant characteristics are presented in Table 1. Most of the participants were females (81%), the mean age was 66.8 ± 5.2 years, and body mass index was 28.0 ± 3.9 kg/m^2^.

**TABLE 1:** Participants’ characteristics.

	All (*n* = 82)	Female (*n* = 66)	Male (*n* = 16)
Age (years)	66 ± 5.2	66 ± 5.1	68 ± 5.4
Hypertension (%)	52.4	53.0	50.0
Diabetes mellitus (%)	24.4	24.3	25.0
Dyslipidaemia (%)	42.7	48.5	18.8
MED BP (%)	52.4	54.6	43.8
MED DM (%)	25.6	22.7	37.5
MED dyslipidaemia (%)	30.5	34.9	12.5
BMI (kg/m^-2^)	28.0 ± 3.9	28.2 ± 4.1	27.2 ± 3.0
DBP (mmHg)	70 ± 8.6	69 ± 8.5	74 ± 7.9
SBP (mmHg)	127 ± 16.8	125 ± 16.4	134 ± 16.9
Fasting glucose (mg/dL)	111 ± 27.8	111 ± 29.1	111 ± 22.2
Total cholesterol (mg/dL)	208 ± 45.9	210 ± 48.2	200 ± 34.9
aPWV (m/s)	9.6 ± 1.0	9.5 ± 1.1	9.9 ± 1.0
cIMT (µm)	774.0 ± 139.0	765.0 ± 133.3	812.0 ± 159.2
MVPA (min/day)	22.0 ± 25.8	19.0 ± 20.4	35.0 ± 39.4
Meeting PA guidelines (%)	35.4	33.3	44.3
6MWT (m)	493 ± 73.7	482 ± 65.2	537 ± 91.2

Data are mean ± standard deviation.

Abbreviations: aPWV, aortic pulse wave velocity; BMI, body mass index; cIMT, carotid intima-media thickness; DBP, diastolic blood pressure; MVPA, moderate-to-vigorous physical activity; SBP: systolic blood pressure; 6MWT, 6-min walking test; MED, medication; DM, diabetes mellitus.

### 3.1 Associations between MVPA and CRF with the vascular health phenotypes

**FIGURE 2:**
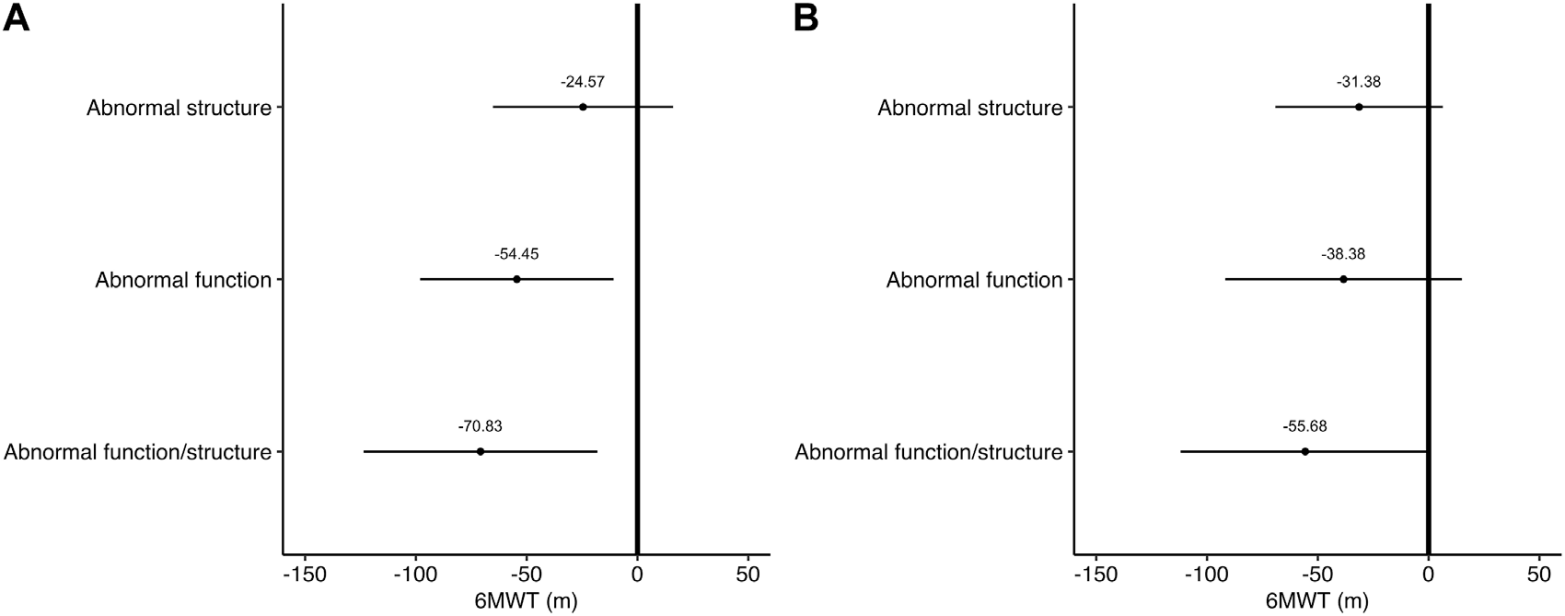
Associations between 6MWT and the vascular phenotypes. Values are model estimates and 95% confidence interval. In **(A)** estimates are adjusted for BMI, and medication for diabetes, hyperlipaemia, and hypertension. In **(B)** estimates are adjusted for BMI, and medication for diabetes, hyperlipaemia, and hypertension, age, sex, systolic, and diastolic blood pressure.

The characteristics of the groups of vascular health phenotypes are presented in Table 2. Figure 2 depicts the association between MVPA and 6MWT with the vascular health phenotypes. In both models, no significant associations between MVPA and the vascular health phenotypes were observed. In contrast, Model 1 showed a linear response between the groups of vascular health phenotypes and the 6MWT. Participants with abnormal aPWV but normal cIMT (β = −53.76; 95% CI = −97.73—−9.78 m; *p* = 0.017), and participants with both abnormal aPWV and cIMT (β = −71.89; 95% CI = −125.46—−18.31 m; *p* = 0.009) had worse 6MWT performance compared with participants with both normal vascular function and structure (healthiest vascular health phenotype). There was no difference in the distance covered in the 6MWT between participants with normal aPWV and abnormal cIMT and participants with both normal vascular function and structure (β = −27.09; 95% CI = −68.28–14.10; *p* = 0.194). Model 2 showed that participants with both abnormal aPWV and cIMT had worse 6MWT performance than participants with both normal vascular function and structure (β = −55.68 95% CI = −111.95–0.59; *p* = 0.052).

**TABLE 2:** Participants’ characteristics according to the vascular health phenotypes.

	Normal structure and function (*n* = 22)	Abnormal structure only (*n* = 29)	Abnormal function only (*n* = 19)	Abnormal structure and function (*n* = 12)
Age (years)	64.7 ± 3.9	63.6 ± 3.5	72.3 ± 4.4	69.9 ± 3.4
BMI (kg/m^2^)	27.2 ± 3.9	29.1 ± 4.4	26.9 ± 3.4	28.8 ± 3.5
DBP (mmHg)	71.0 ± 8.0	70.5 ± 8.5	70.6 ± 8.9	67.3 ± 10.2
SBP (mmHg)	121.3 ± 15.7	121.6 ± 12.7	138.0 ± 16.0	134.1 ± 19.6
Glucose (mg/dL)	105.6 ± 16.1	105.6 ± 16.7	120.5 ± 42.6	120.0 ± 34.0
Total cholesterol (mg/dL)	205.0 ± 55.1	219.2 ± 42.6	211.1 ± 38.8	184.9 ± 41.4
HDL (mg/dL)	44.8 ± 8.9	48.1 ± 12.8	45.9 ± 13.0	37.8 ± 9.7
MVPA (min/day)	33.3 ± 36.8	18.5 ± 17.7	19.9 ± 23.8	15.6 ± 16.7
6MWT (m)	533.9 ± 78.0	494.7 ± 57.9	474.2 ± 69.3	444.3 ± 74.4
cIMT (µm)	635.6 ± 65.4	864.7 ± 113.1	718.4 ± 82.5	900.0 ± 88.2
aPWV (m/s)	9.0 ± 0.6	8.9 ± 0.5	10.8 ± 0.8	10.7 ± 0.7

Data are mean ± standard deviation. BMI: body mass index; DBP: diastolic blood pressure; SBP: systolic blood pressure; MVPA: moderate-to-vigorous physical activity; 6MWT: 6-min walking test; cIMT: carotid intima-media thickness; aPWV: aortic pulse wave velocity.

### 3.2 Associations between MVPA and CRF with aPWV and cIMT

**FIGURE 3:**
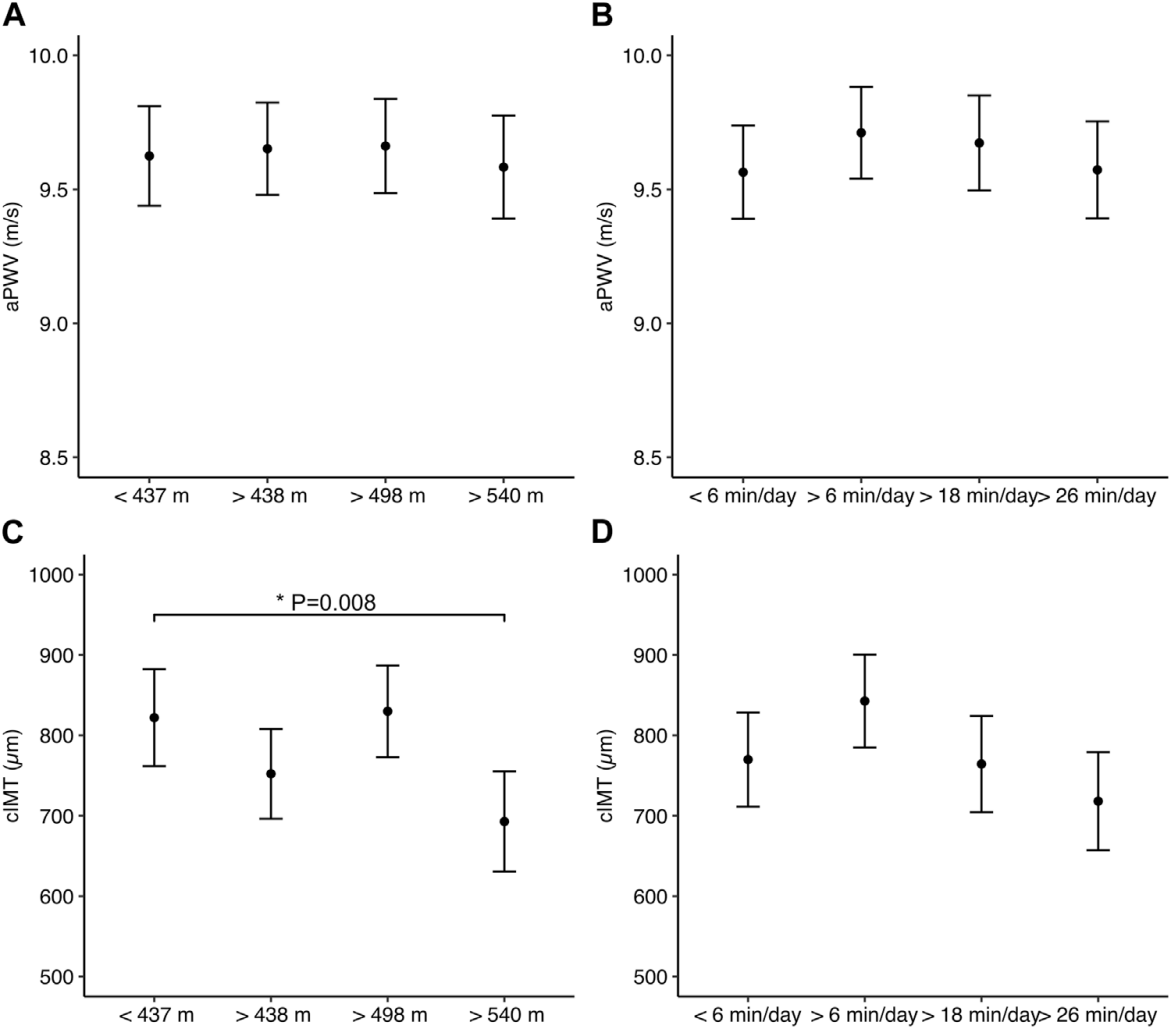
Associations between moderate-to-vigorous physical activity (right panel) and 6-min walk test (left panel) with markers of vascular function **(A**and **B)** and structure **(C** and **D)**. Values are model estimates and error bars represent the 95% confidence interval. Estimates are adjusted for BMI, and medication for diabetes, hyperlipaemia, and hypertension, age, sex, systolic, and diastolic blood pressure.

After controlling for confounders, no association was observed between MVPA and aPWV (β = 0.001; 95% CI = −0.0001–0.001 m/s; *p* = 0.838) or between MVPA and cIMT (β = −0.75; 95% CI = −2.04–0.54 µm; *p* = 0.250). No association was found between the 6MWT and aPWV (β = −0.001; 95% CI = −0.0001–0.001 m/s; *p* = 0.908). However, a linear association was observed between the 6MWT and cIMT (β = −0.58; 95% CI = −1.07−0.10 m/s; *p* = 0.019 µm). The dose-response association between 6MWT quartiles, aPWV, and cIMT is shown in Figure 3. No dose-response association was observed between the 6MWT and aPWV. In contrast, older adults who walked more than 540 m had thinner cIMT (β = −124.84; 95% CI = −220.22−29.46 µm; *p* = 0.011) than participants who covered <437 m. No dose-response association was observed between MVPA and aPWV or cIMT (all *p* > 0.05).

## 4 Discussion

This study investigated the associations between MVPA and CRF with vascular health phenotypes, including the combined measures of aPWV and cIMT, in older adults. The main findings were: 1) MVPA was not associated with any vascular measure either alone or combined into the vascular health phenotypes; 2) lower 6MWT performance was observed in participants with both abnormal aPWV and cIMT; and 3) participants with the highest 6MWT performance (>540 m) had thinner cIMT than their unfit peers (<437 m). Taken together, our findings add novel data to the literature showing that low CRF, but not low MVPA, was associated with the unhealthiest vascular health phenotype (abnormal aPWV/cIMT) in older adults.

The main novelty of the present study is the comparison of MVPA and 6MWT performance among the different groups of vascular health phenotypes (Figure 2). For this, we used values of aPWV and cIMT, which are positively linked with cardiovascular disease morbidity and mortality (Lorenz et al., 2007; Vlachopoulos et al., 2010) and are also advocated by different consensus statements (Stein et al., 2008; Van Bortel et al., 2012). Values above 10 m/s were defined as increased aPWV in accordance with the consensus of the European Society of Hypertension, which stipulates this as a standard value to use in clinical practice (Van Bortel et al., 2012). Furthermore, a recent meta-analysis provided evidence that 10 m/s is associated with cardiovascular and all-cause mortality (Sequí-Domínguez et al., 2020). When compared to reference values published using the same aPWV assessment as in our present investigation, the cut-off of 10 m/s for aPWV falls within the range of the 75th percentile for sex, age, and the presence of cardiovascular disease risk factors (e.g., 9.7–12.9 m/s) (Paiva et al., 2020), indicating that our choice is in agreement with the increased aPWV in the population. Similarly, we followed the Brazilian Society of Cardiology to define increased cIMT and the Consensus Statement from the American Society of Echocardiography (Stein et al., 2008). Both societies specify the sex, age, and race 75th percentile as the cut-off for defining increased cIMT, and we utilised previously published population-specific values (Santos et al., 2014). However, it should also be pointed that combining participants into four groups of vascular health phenotypes decreased the sample size with 12 participants presenting in the unhealthiest vascular phenotype, although previous studies have investigated similar sample sizes (Kobayashi et al., 2004; Tzortzis et al., 2010; Nagai et al., 2013).

Combining aPWV and cIMT builds on previous studies that have demonstrated an improved prediction of cardiovascular disease events when combining of these markers together (Kobayashi et al., 2004; Tzortzis et al., 2010; Nagai et al., 2013). For instance, Nagai et al. (2012) showed that participants with both increased aPWV and cIMT had an odds ratio of 4.9 for vascular events in a follow-up of ∼2 years, which was significantly higher than that of participants with only one of these markers impaired. Similarly, Tzortzis et al. (2010) found that adults with abnormal aPWV and cIMT had significantly higher odds (11.2) of poorer cardiac function than those with aPWV (odds ratio, 5) or cIMT (odds ratio, 3.5) alone. These findings indicate that having both abnormal aPWV and cIMT significantly increases the odds of cardiovascular disease, and strategies to prevent a worsened vascular health phenotype are desired. Our results indicate that older adults with both abnormal aPWV and cIMT covered approximately 50 m less distance in the 6MWT. This suggests that maintaining adequate CRF levels should be considered in older adults to maintain adequate vascular health, although future investigations are needed to establish a possible cause-effect relationship. Notably, 50 m has been advocated as the minimum threshold for improvements in the 6MWT performance in older adults (Bouaziz et al., 2018). Future investigations should include larger sample sizes and investigate possible physiological determinants with direct assessment of CRF (e.g., VO_2_max).

Comparable with the findings showing an association between VO_2_max and arterial stiffness (Jae et al., 2022), our results suggest that CRF is also associated with overall vascular health, i.e., the vascular health phenotypes including the combination of aPWV and cIMT. Notably, associations were still observed (though borderline with *p* = 0.052) even after controlling for BP and age, which are strong determinants of aPWV (Paiva et al., 2020). This suggests that improving CRF is an important therapeutic strategy for older adults with increased cardiovascular disease risk, given that participants with lower 6MWT performance had the unhealthiest vascular health phenotype, characterised by abnormal aPWV and cIMT. One potential physiological mechanism for this association is improved nitric oxide vasodilatory capacity of the arterial wall, as observed in previous studies (Deiseroth et al., 2019). This high vessel response to vasodilatory stimulus may be mediated by a reduction in inflammatory markers associated with ageing (Santos-Parker et al., 2014). Future studies using other measures of vascular function, such as flow-mediated dilation (FMD) and assessment of microvascular function, could further elucidate the mechanisms underlying the protective effects of CRF on vascular health in older adults.

The volunteers in the present study covered a distance in the 6MWT corresponding to the 10–25th percentile (310–545 m) of normative data based on community-dwelling American older adults (Rikli and Jones, 1998). Compared to other studies, older adults covered distances between 344–613 m (Troosters et al., 1999). Differences in the total distance covered between studies are likely explained by different sample characteristics and study protocols, such as the type of course used for testing and participant familiarisation. Moreover, female participants and older individuals tend to cover less distance in the walking test (Morales-Blanhir et al., 2011), which may explain the values obtained in the present study. The 6MWT was used to measure CRF, and our findings add to the existing literature showing an inverse association between CRF and arterial stiffness across lifespan (Boreham et al., 2004; Veijalainen et al., 2016; Haapala et al., 2020). This highlights the important role of CRF in maintaining cardiovascular health.

Because it is easy to apply in a clinical setting, the present findings emphasise that the 6MWT can be an important tool for cardiovascular disease screening in older adults. For example, participants in the present investigation who covered distances of 540 m had cIMT that was 125 µm thinner than that of participants who covered <437 m. This difference between quartiles may be of clinical relevance given that increases in cIMT are associated with CVD mortality (Vlachopoulos et al., 2010). Lee et al. (2021) have shown an inverse association between cIMT and the estimated VO_2_max in middle-aged and older adults. Importantly, it has been suggested that decreases in arterial function may occur before changes in arterial structure occur (Costantino et al., 2016), with studies indicating that arterial function predicts cIMT remodelling in longitudinal investigations (Thijssen et al., 2016). Therefore, our findings suggest a window of opportunity for delivery interventions designed to improve arterial function before structural changes occur.

Although a higher CRF was associated with better markers of vascular health, we did not observe significant associations between MVPA, aPWV, and cIMT. Our observational findings add to Tanaka et al., (2002) who demonstrated no significant changes in cIMT following a 3-month period of an exercise intervention at 60% of the maximum heart rate in older adults. Similarly, we found no associations between MVPA and arterial function in our study, which is consistent with the results of Caviezel et al. (2015), who demonstrated no association between MVPA and carotid distensibility. However, contrary to our findings, a meta-analysis has evidenced an inverse association between PA levels and aPWV. Germano-Soares et al. (2018) included articles with participants with different characteristics, such as age range, CVD comorbidities, and PA levels, which may explain the different results. Furthermore, as our studied sample consisted mostly of overweight women, our findings align with those of Stamatelopoulos et al. (2020), who reported that objectively measured physical activity was not significant correlated with aPWV in older overweight women. Similarly, Gomez-Marcos et al. (2014) found no association between PA and aPWV or cIMT in a sample of adults after controlling for confounders. Overall, our findings, and those of others, indicate that debate still exists on whether PA levels impact arterial function and structure in older adults.

Although the literature indicates that ∼10 and ∼17 min/day of MVPA may reduce the risk of CVD (Silva et al., 2022), our results show that accumulating >27 min/day of MVPA was not associated with better aPWV and cIMT in older adults (Figure 3). On average, male and female participants performed 35 ± 39.4 and 19 ± 20.4 min/day of MVPA, with 44% and 33% of them meeting current PA guidelines (Izquierdo et al., 2021). Additionally, enormous variation around the mean was present, which is normal in studies assessing PA levels (dos Santos et al., 2020). It is worth highlighting that although different cut-offs have been published to determine MVPA levels (dos Santos et al., 2020), the objectively measured MVPA in the present study ensured a robust PA assessment. The lack of association between MVPA and vascular health markers contradicts our initial hypothesis and previous studies (Park et al., 2017; Germano-Soares et al., 2018). Several reasons may explain these findings, such as the amount and pattern of MVPA levels, and the skewed nature of PA with few individuals achieving high while a vast majority achieve lower MVPA levels, although logarithm-adjusting MVPA in the models did not change our findings. Intervention studies controlling for the intensity, duration, type, and frequency of the exercise stimuli would clarify whether exercise or CRF is required to improve markers of vascular health, including arterial function and structure.

### 4.1 Strengths and limitations

The present study had several strengths worth highlighting. First, PA levels were objectively measured, with a focus on MVPA, as previous studies have indicated that this intensity range to has consistent health benefits compared with light-intensity PA in older adults (Park et al., 2017). Second, both vascular function and structure were assessed and a combination of these outcomes was used to create vascular health phenotypes. Finally, the 6MWT was used in the present investigation, which is an easy-to-implement test in clinical practice and is recommended by the American Health Association (ATS Committee on Proficiency Standards for Clinical Pulmonary Function Laboratories, 2002) to assess CRF. Values < 437 m could serve as a “red flag” to indicate poor vascular health in older adults, characterised by abnormal aPWV and cIMT.

However, the present investigation has some limitations. First, the cross-sectional design precludes the establishment of causality, although it is unlikely a poor vascular function or structure would lead to low CRF or MVPA levels in older adults. Future longitudinal and/or interventional investigations would help to clarify the direction of the associations observed in our study. Second, as shown in Figure 1, sample losses occurred due to late initiation of cIMT assessment, as well as a high number of participants lacking objective assessment of PA. Finally, while we recruited community-dwelling older adults using diverse advertisement methods, healthy volunteer bias may have been an issue, and sex-specific bias is limited due to the predominance of female participants.

## 5 Conclusion

Our data suggest that poorer CRF, but not lower MVPA, is associated with the unhealthiest vascular health phenotype (abnormal aPWV/cIMT) in older adults. Therefore, the assessment of CRF seems useful in identifying those older adults with combined abnormal vascular function and structure, who may have a higher risk of future adverse cardiovascular events.

## Data Availability

The raw data supporting the conclusion of this article will be made available by the authors, without undue reservation.
